# Accuracy of Three Different Strategies for Virtual Interocclusal Registration: An In Vitro Study

**DOI:** 10.1111/jerd.70213

**Published:** 2026-07-06

**Authors:** Ragai Edward Matta, Felix Förtsch, Susanne Pfeuffer, Manfred Wichmann, Johannes Ries

**Affiliations:** ^1^ Department of Prosthodontics (Head: Prof. Dr. Manfred Wichmann), Glueckstrasse 11, 91054 Erlangen Erlangen University Hospital, Friedrich‐Alexander‐University Erlangen‐Nuremberg Erlangen Germany

**Keywords:** digital workflow, intraoral scanner, maxillomandibular relationship, virtual articulation, virtual interocclusal registration

## Abstract

**Objective:**

The aim of this study was to compare the accuracy of three different strategies for virtual interocclusal registration in a fully dentate in vitro model.

**Materials and Methods:**

Three strategies for virtual interocclusal registration were compared: bilateral buccal registration, unilateral buccal registration, and anterior registration. For this purpose, two fully dentate resin models articulated using average‐value settings were digitized 15 times per strategy using three different intraoral scanners. Accuracy was evaluated by superimposition of the intraoral scans with a virtual reference file generated using an industrial high‐precision scanner.

**Results:**

Regardless of the intraoral scanner used, bilateral buccal registration demonstrated significantly higher accuracy in terms of mean deviation across the entire dental arch compared with the other two strategies. No consistent trend was observed between unilateral buccal and anterior registration. For both unilateral buccal and anterior registration, a decrease in precision was observed with increasing distance from the registration site.

**Conclusions:**

In fully dentate patients, bilateral buccal registration provides the highest accuracy for maxillomandibular alignment.

**Clinical Significance:**

A precise determination of the maxillomandibular relationship is essential in restorative and esthetic dentistry to ensure accurate model analysis, treatment planning, and fabrication of restorations.

**Abbreviations**
AR: Anterior registration
BBR: Bilateral buccal registration
UBR: Unilateral buccal registration
VIR: Virtual interocclusal registration

## 1 Introduction

With the ongoing digitalization of dentistry, workflows in prosthetic treatment are undergoing substantial changes. Whereas treatment previously relied on conventional procedures such as traditional impression taking, the fabrication of gypsum casts, and the production of prosthetic restorations using the lost‐mold technique, patients can now be treated using fully digital workflows. In this approach, intraoral scanners are employed for impression taking, followed by computer‐assisted design and manufacturing of the prosthetic restoration [1, 2]. Accurate impressions form the basis for a well‐fitting restoration and thus determine the overall success of prosthetic treatment [3, 4]. The accuracy of digital impressions obtained via intraoral scanners has been extensively investigated, and several reviews have concluded that the digital method yields results comparable to or even superior to those of conventional impressions [5, 6, 7]. Moreover, digital impression taking offers several advantages, including increased patient comfort, savings in time and cost, and consequently improved clinical efficiency [8, 9].

In addition to the accuracy of the digital models generated by intraoral scanning, the precision with which these models are aligned with one another plays a critical role. The accuracy of virtual interocclusal registration (VIR) has already been investigated in numerous studies. Recent systematic reviews on this topic concluded that the accuracy of VIR using intraoral scanners is comparable to, or even superior to, that of conventional methods [10, 11, 12, 13, 14]. Of particular note is the excellent reproducibility reported for VIR compared with traditional maxillomandibular relationship registration [15, 16, 17].

Various procedures and techniques for performing VIR have been described, and a limited number of comparative studies have examined these different approaches. However, because these studies used heterogeneous methods, the current evidence base is not yet sufficient to support well‐founded clinical recommendations. Two studies identified a bilateral buccal registration as the most accurate technique [18, 19], whereas another study comparing anterior and unilateral buccal registration reported that anterior registration yielded more precise results [20]. Therefore, the aim of the present study was to compare different strategies for VIR using an intraoral scanner and to expand the available evidence in order to provide clear recommendations for clinical practice. For this study, three VIR strategies were compared: bilateral buccal registration, unilateral buccal registration, and anterior registration. Intraoral scans were obtained from fully dentate, articulated resin models and subsequently superimposed and compared with a reference model generated using an industrial high‐precision scanner to evaluate accuracy in a digital analysis software.

The tested research hypotheses were as follows: (1) Bilateral buccal registration provides the highest accuracy for maxillomandibular relationship recording and (2) anterior registration yields more accurate VIR results than unilateral buccal registration.

## 2 Materials and Methods

Two fully dentate resin models were fabricated and mounted in an articulator according to average values (KaVo PROTARevo 7, KaVo Dental GmbH, Biberach, Germany). A minimal adjustment of the occlusion was performed to achieve a uniform occlusal contact, ensuring at least one contact point per tooth in the posterior region. To compare different strategies for VIR, three approaches were selected: unilateral buccal registration on the left side (UBR), bilateral buccal registration (BBR), and anterior registration (AR). For buccal registrations, scanning was performed from the first premolar to the first molar; for anterior registrations, the scan extended from canine to canine. Three intraoral scanners were used for digital impression taking: Primescan (Dentsply Sirona, Charlotte, USA), Trios 4 (3Shape, Copenhagen, Denmark), and iTero Element (Align Technologies, San Jose, USA). For each scanner, after digitizing the baseline models according to manufacturer recommendations, VIR was performed using each of the three selected methods. Each registration was repeated 15 times per scanner and method. The number of repetitions was selected in accordance with recent comparative studies, which also performed 10 to 15 repetitions per experimental group [12, 18, 19]. To obtain the most robust and meaningful arithmetic mean possible, we chose a sample size of *n* = 15 per strategy. A load of 50 N was applied to the articulator during VIR. All resulting digital models were exported as STL files for analysis. Subsequent data analysis was performed using evaluation software (ATOS Professional 2018, Carl Zeiss GOM Metrology GmbH, Braunschweig, Germany). For this purpose, the master models were first digitized individually as well as in occlusion using an industrial high‐precision scanner (ATOS So4 II, Carl Zeiss GOM Metrology GmbH) in order to generate paired virtual reference models. A coordinate system with its origin at the center of the occlusal plane was defined for each mandibular tooth (37–47) in the virtual reference model. Thereafter, all intraoral scans were imported and analyzed individually. Initially, the articulated comparison models were superimposed using the lower jaw. Subsequently, the coordinate systems of the mandibular reference model were transferred to the corresponding mandibular comparison model. Finally, a best‐fit superimposition was performed on the maxillary model, allowing deviations between the respective coordinate systems of the mandibular reference and comparison models to be recorded. The mean deviation across the entire dental arch was calculated, and differences between the various regions of the dentition were subsequently compared. For this purpose, the dentition was divided into an anterior region (FDI 33–43) and two posterior regions (FDI 37–34 and 44–47). Statistical analyzes were performed using the R program (version 4.5.2; R Core Team, R Foundation for Statistical Computing, Vienna, Austria), with the level of significance set at *α* = 0.05. For the evaluation of deviations across the entire dental arch, a two‐way analysis of variance (ANOVA) was initially conducted to assess the effects of the VIR strategy and the intraoral scanner used. For comparisons of the individual registration methods across different dental regions, as well as comparisons among dental regions within each registration method, a three‐way ANOVA was applied accordingly. Subsequently, post hoc tests were performed conditional on the remaining variables, and *p*‐values were adjusted using the Holm correction method. For the mean deviation across the entire dental arch, *p*‐value adjustment was performed across all values within each scanner. For deviations in the individual dental regions, *p*‐value adjustment was performed across all values within each scanner–method combination and within each scanner–region combination. An overview of the study design is shown in Figure 1.

**FIGURE 1:**
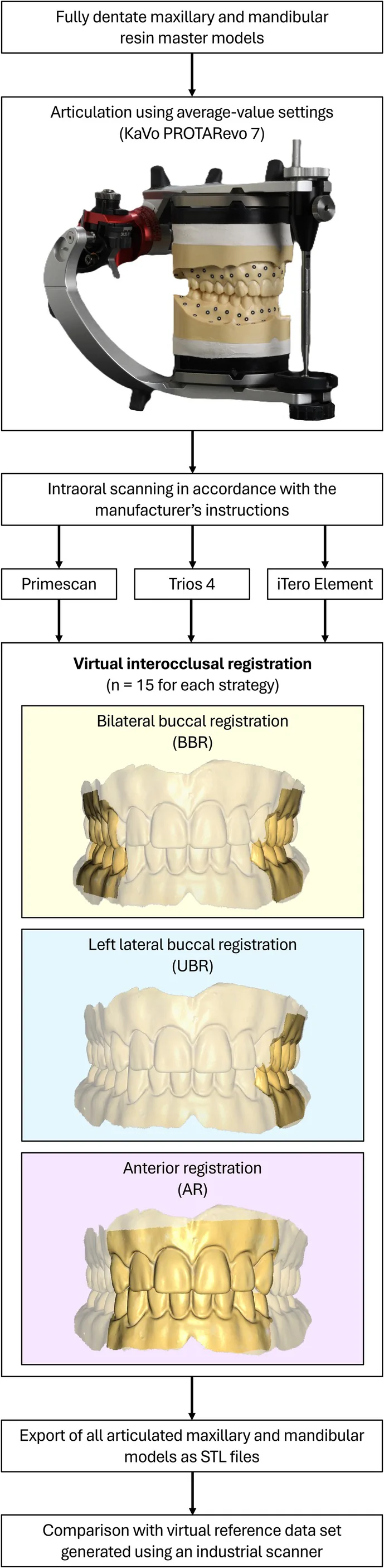
Overview of the study design.

## 3 Results

For the deviation across the entire dental arch, the two‐factor ANOVA revealed a significant effect of both the VIR strategy (*p* < 0.001) and the intraoral scanner (*p* < 0.001) used, as well as a significant interaction between the two variables (*p* < 0.001). Therefore, post hoc tests were performed to compare the VIR strategies depending on the intraoral scanner used. BBR exhibited significantly lower deviations than the other two methods, irrespective of the intraoral scanner used (Tables 1 and 2, Figure 2). In the comparison between UBR and AR, AR demonstrated higher accuracy for the Trios 4 and iTero Element scanners, although this difference reached statistical significance only for the Trios 4 scanner. In contrast, for the Primescan scanner, UBR proved to be significantly more accurate.

**TABLE 1:** Descriptive statistics of the mean spatial deviation across the entire dental arch: Mean deviation (Mean) with standard deviation (SD) and the minimum (Min) and maximum (Max) deviation.

	Mean ± SD [Min–Max] μm		
	BBR	UBR	AR
Primescan	29 ± 12 [7–72]	76 ± 45 [13–266]	112 ± 101 [7–429]
Trios 4	62 ± 31 [14–173]	187 ± 109 [58–559]	142 ± 108 [6–582]
iTero Element	52 ± 23 [8–144]	77 ± 58 [3–296]	68 ± 36 [22–234]

**TABLE 2:** *p* values for all statistical comparisons of the mean spatial deviations across the entire dental arch.

	BBR versus UBR	BBR versus AR	UBR versus AR
Primescan	*p* < 0.001	*p* < 0.001	*p* < 0.001
Trios 4	*p* < 0.001	*p* < 0.001	*p* < 0.001
iTero Element	*p* < 0.001	*p = 0.027*	*p* = 0.181

**FIGURE 2:**
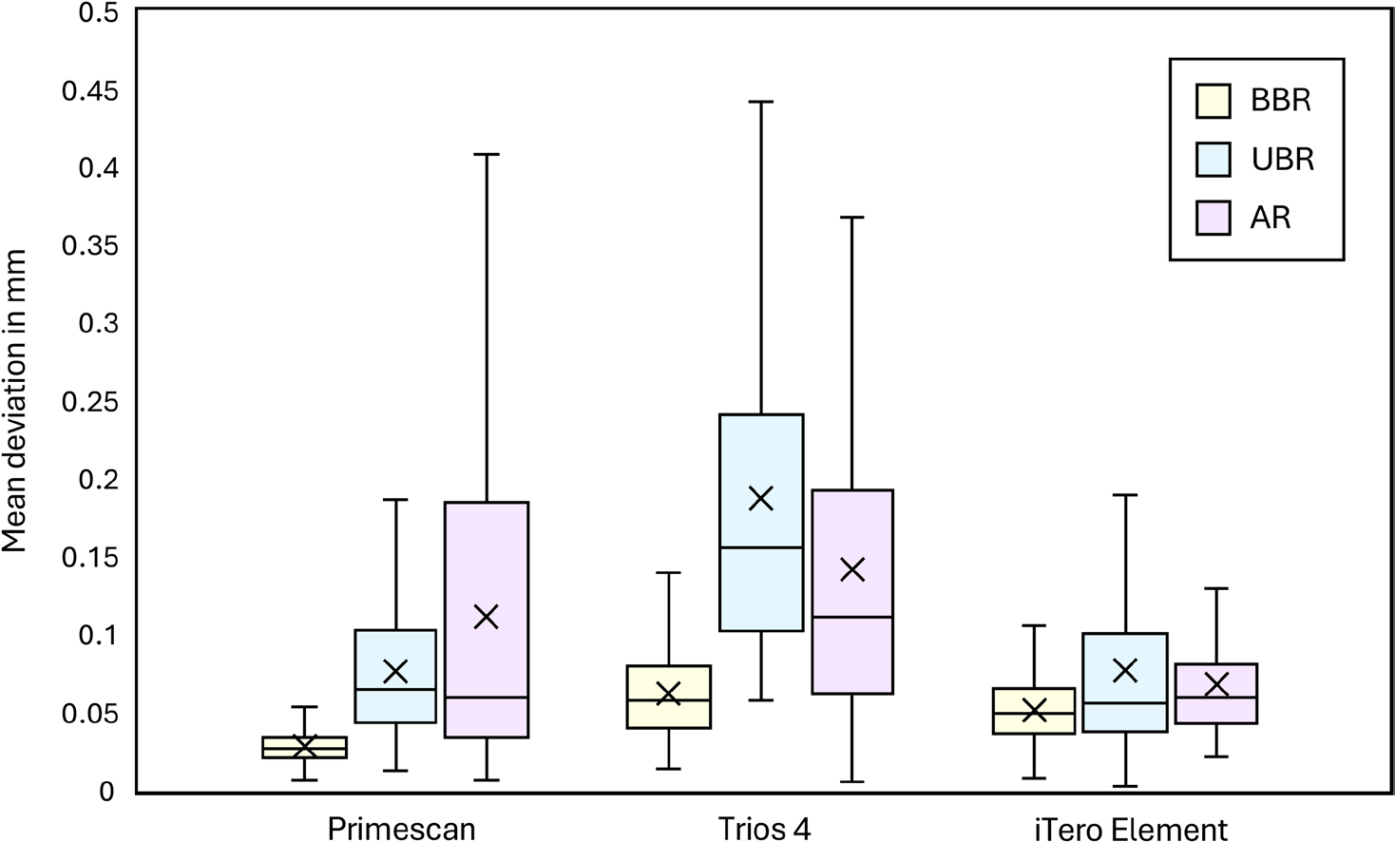
Box‐whisker‐plot showing the mean spatial deviation across the entire dental arch for all VIR methods.

In addition to deviations across the entire dental arch, deviations within the different dental regions were also analyzed and compared. Here, the three‐factor ANOVA revealed a significant effect of the VIR strategy, dental region, and intraoral scanner (*p* < 0.001), as well as a statistically significant interaction among all variables (*p* < 0.001). Across all dental regions, BBR consistently demonstrated the highest accuracy, with the majority of comparisons being statistically significant (Tables 3 and 4).

**TABLE 3:** Descriptive statistics of the mean spatial deviation across the different dentition regions: Mean deviation (Mean) with standard deviation (SD) and the minimum (Min) and maximum (Max) deviation for the tooth segments 37–34, 33–43, and 44–47.

		Mean ± SD [Min–Max] μm		
		BBR	UBR	AR
Primescan	37–34	30 ± 11 [8–58]	50 ± 27 [13–165]	189 ± 105 [22–429]
	33–43	26 ± 12 [9–57]	73 ± 31 [17–155]	35 ± 13 [7–82]
	44–47	31 ± 12 [7–72]	108 ± 56 [15–266]	149 ± 92 [21–385]
Trios 4	37–34	79 ± 28 [24–173]	106 ± 40 [58–220]	222 ± 112 [33–582]
	33–43	60 ± 31 [14–144]	165 ± 73 [65–377]	66 ± 34 [12–140]
	44–47	48 ± 25 [16–140]	302 ± 110 [117–559]	174 ± 105 [6–480]
iTero Element	37–34	40 ± 19 [8–83]	52 ± 26 [15–122]	78 ± 36 [25–192]
	33–43	57 ± 20 [16–102]	67 ± 41 [3–190]	51 ± 17 [22–100]
	44–47	55 ± 26 [10–144]	117 ± 79 [13–296]	84 ± 45 [25–234]

**TABLE 4:** *p* values for all statistical comparisons of the mean spatial deviations of the different registration methods in the different regions of the dentition.

		BBR versus UBR	BBR versus AR	UBR versus AR
37–34	Primescan	*p* = 0.037	*p* < 0.001	*p* < 0.001
	Trios 4	*p* = 0.006	*p* < 0.001	*p* < 0.001
	iTero Element	*p* = 0.235	*p* < 0.001	*p* = 0.015
33–43	Primescan	*p* < 0.001	*p* = 0.249	*p* < 0.001
	Trios 4	*p* < 0.001	*p* = 0.441	*p* < 0.001
	iTero Element	*p* = 0.428	*p* = 0.429	*p* = 0.126
44–47	Primescan	*p* < 0.001	*p* < 0.001	*p* < 0.001
	Trios 4	*p* < 0.001	*p* < 0.001	*p* < 0.001
	iTero Element	*p* < 0.001	*p* = 0.002	*p* = 0.001

Furthermore, accuracy within the different regions of the dentition was compared for each registration method individually. No clear trend emerged for BBR; however, the other two methods produced distinct and scanner‐independent patterns. For AR, significantly lower deviations were observed in the anterior region compared with both posterior regions. For UBR performed on the left side, accuracy decreased significantly with increasing distance from the registration site: the left posterior region showed significantly higher accuracy than the other two regions, and the anterior region was significantly more accurate than the right posterior region (Table 5, Figure 3).

**TABLE 5:** *p* values for all intramethod statistical comparisons of the mean spatial deviations across the different regions of the dentition.

		37–34 versus 44–47	37–34 versus 33–43	44–47 versus 33–43
BBR	Primescan	*p* = 1	*p* = 1	*p* = 1
	Trios 4	*p* = 0.004	*p* = 0.063	*p* = 0.172
	iTero Element	*p* = 0.285	*p* = 0.174	*p* = 0.772
UBR	Primescan	*p* < 0.001	*p* = 0.012	*p* < 0.001
	Trios 4	*p* < 0.001	*p* < 0.001	*p* < 0.001
	iTero Element	*p* < 0.001	*p* = 0.088	*p* < 0.001
AR	Primescan	*p* < 0.001	*p* < 0.001	*p* < 0.001
	Trios 4	*p* < 0.001	*p* < 0.001	*p* < 0.001
	iTero Element	*p* = 0.52	*p* = 0.005	*p* < 0.001

**FIGURE 3:**
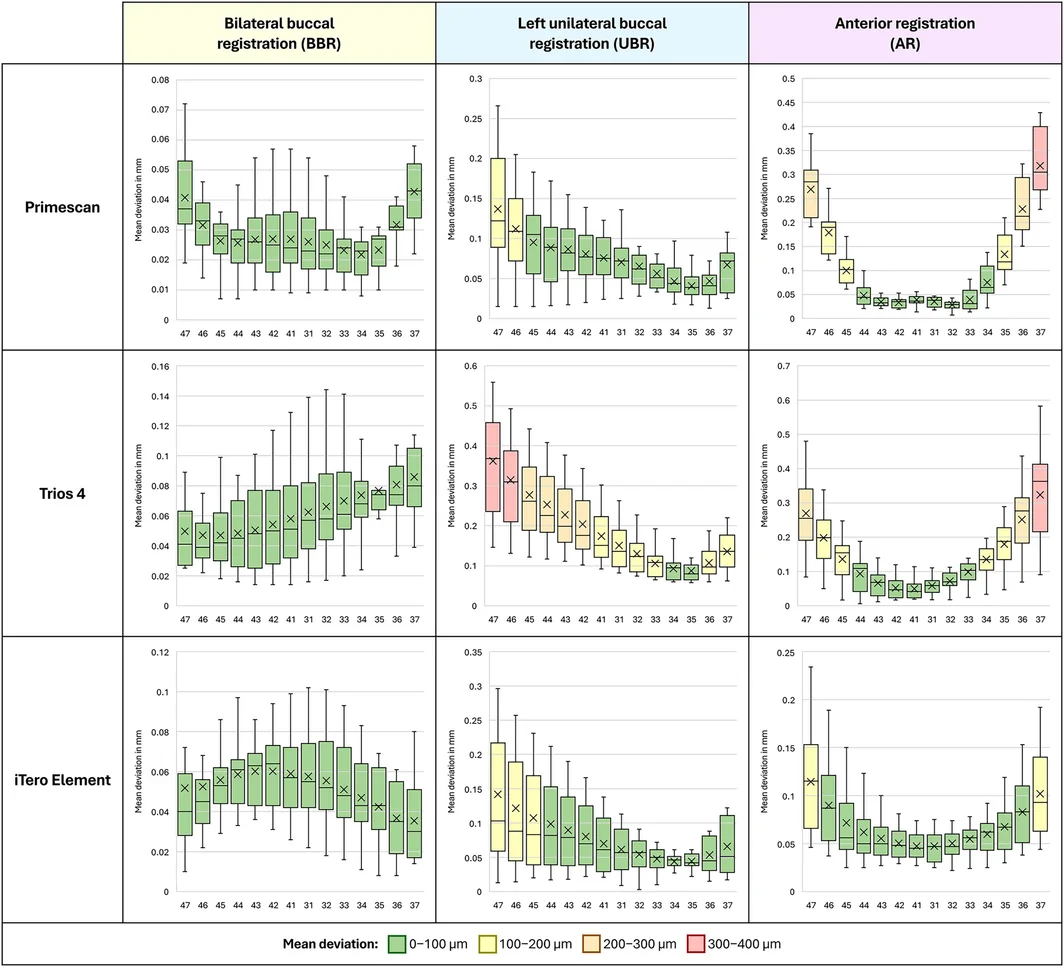
Box‐whisker‐plots showing the mean spatial deviations for every tooth for all registration methods and intraoral scanners used.

## 4 Discussion

The present in vitro study investigated the influence of different approaches on the accuracy of virtual interocclusal registration (VIR) in fully dentate patients. The results clearly demonstrated that the scanning strategy used for VIR has a significant impact on the accuracy of maxillomandibular alignment. When assessing mean accuracy across the entire dental arch, BBR proved to be significantly more accurate than the other two methods, regardless of the intraoral scanner used. In contrast, the comparison between UBR and AR did not yield a definitive conclusion: with the Primescan, UBR produced significantly higher accuracy, whereas with the Trios 4, AR showed significantly better results. Using the iTero scanner, no significant difference was found between the two approaches. These findings support the interpretation that the spatial distribution of the scanned registration areas is a relevant determinant of VIR accuracy. Bilateral posterior registration may provide a broader geometric constraint for maxillomandibular alignment, whereas unilateral or anterior registration may permit distance‐dependent deviations away from the registration site.

These findings align with the results of two previous comparative studies that also investigated different VIR strategies and identified BBR as the most precise method [18, 19]. One study compared full‐arch VIR with a bilaterally segmented approach involving entire quadrants and found the segmented method to be significantly more accurate [18]. The other comparative study evaluated various VIR strategies and likewise reported that the segmented approach involving full quadrants was more precise than full‐arch registration. This study also assessed all methods investigated in the present study and similarly found bilateral buccal registration in the posterior region to yield significantly higher accuracy than the other approaches. Moreover, bilateral posterior registration proved significantly more accurate than the bilateral approach involving complete quadrants. As in the present study, the comparison between UBR and AR produced no clear superiority of either method, underscoring the consistency of the findings. The deviations reported in this comparative study (BBR: 59 μm; UBR: 70 μm; AR: 79 μm) are of the same magnitude as those observed in the present investigation. Anterior registration was also performed from canine to canine, while the posterior scanning region extended from the first to the second molar rather than from the first premolar to the first molar [19]. This methodological consistency, along with the concordant results, reinforces the validity and reliability of the findings of the present study. Thus, while previous comparative studies have suggested the relevance of the distribution of scanned registration surfaces [18, 19], this aspect has not, to our knowledge, been evaluated in the same combined experimental setting; the present study therefore extends this evidence by testing BBR, UBR, and AR within a single standardized fully dentate in vitro design across three IOS systems and by using an industrial high‐precision reference workflow with coordinate‐based regional analysis to characterize overall and distance‐dependent accuracy patterns.

In addition to evaluating deviations across the entire dental arch, the methods were also compared within specific regions of the dentition. As observed in the overall arch comparison, BBR again proved to be the most accurate method across all regions, with the sole exception that AR demonstrated significantly higher accuracy in the anterior region when using the iTero scanner. Beyond comparing the different methods, accuracy within each dentition region was also assessed for each registration technique. For AR, all intraoral scanners consistently showed significantly greater accuracy in the anterior region compared with the posterior regions. In unilateral buccal registration, the side on which registration was performed was significantly the most precise, and the anterior region was significantly more accurate than the contralateral posterior region. Thus, UBR exhibited a progressive decline in accuracy with increasing distance from the registration site. This observation is supported by a comparative study that contrasted AR with UBR and likewise found that UBR achieved its highest accuracy on the registration side [20]. Despite this interesting and informative outcome, its clinical relevance remains limited, as BBR consistently proved to be the most precise method in all regions of the dentition, irrespective of the intraoral scanner used.

Despite the insightful findings, the limitations of the present investigation must be acknowledged. Most notably, the in vitro design does not account for patient‐specific challenges encountered during digital impression taking. In clinical situations, cheek retraction, limited exposure of buccal surfaces, saliva, restricted access, and the maintenance of stable occlusal contact may affect the amount and quality of registration data available for alignment, thereby limiting clinical transferability. Furthermore, the intraoral scanners selected represent only a small portion of the systems available on the market.

For fully dentate patients, current evidence supports the use of BBR when sufficient bilateral posterior registration areas are clinically accessible and stable occlusal contact can be maintained. However, regarding clinical applicability and optimal scan strategies in partially dentate patients, additional research is required to establish definitive guidelines.

## 5 Conclusions

Within the limitations of this current study, it was concluded that:

1. In fully dentate patients, bilateral buccal registration provides the highest accuracy for maxillomandibular alignment.
2. When assessing accuracy across the entire dental arch, no clear trend emerged in the comparison between anterior registration and unilateral buccal registration.
3. When anterior registration or unilateral buccal registration is used, accuracy decreases progressively with increasing distance from the registration site.

## Author Contributions

**Johannes Ries:** conceptualization. **Ragai Edward Matta** and **Johannes Ries:** methodology. **Felix Förtsch:** software. **Ragai Edward Matta**, **Johannes Ries**, and **Felix Förtsch:** validation. **Ragai Edward Matta:** formal analysis. **Susanne Pfeuffer** and **Felix Förtsch:** investigation. **Manfred Wichmann:** resources. **Susanne Pfeuffer** and **Felix Förtsch:** data curation. **Felix Förtsch:** writing – original draft preparation. **Ragai Edward Matta** and **Johannes Ries:** writing – review and editing. **Felix Förtsch:** visualization. **Ragai Edward Matta:** supervision. **Manfred Wichmann:** project administration. **Ragai Edward Matta:** funding acquisition. All authors have read and agreed to the published version of the manuscript.

## Funding

The authors have nothing to report.

## Conflicts of Interest

The authors declare no conflicts of interest.

## Data Availability

The data that support the findings of this study are available from the corresponding author upon reasonable request.
